# Central gene expression changes associated with enhanced neuroendocrine and autonomic response habituation to repeated noise stress after voluntary wheel running in rats

**DOI:** 10.3389/fphys.2013.00341

**Published:** 2013-11-25

**Authors:** Sarah K. Sasse, Tara J. Nyhuis, Cher V. Masini, Heidi E. W. Day, Serge Campeau

**Affiliations:** Department of Psychology and Neuroscience, University of ColoradoBoulder, CO, USA

**Keywords:** audiogenic stress, exercise, habituation, glucocorticoid, cardiovascular, *in situ* hybridization

## Abstract

Accumulating evidence indicates that regular physical exercise benefits health in part by counteracting some of the negative physiological impacts of stress. While some studies identified reductions in some measures of acute stress responses with prior exercise, limited data were available concerning effects on cardiovascular function, and reported effects on hypothalamic-pituitary-adrenocortical (HPA) axis responses were largely inconsistent. Given that exposure to repeated or prolonged stress is strongly implicated in the precipitation and exacerbation of illness, we proposed the novel hypothesis that physical exercise might facilitate *adaptation* to repeated stress, and subsequently demonstrated significant enhancement of both HPA axis (glucocorticoid) and cardiovascular (tachycardia) response habituation to repeated noise stress in rats with long-term access to running wheels compared to sedentary controls. Stress habituation has been attributed to modifications of brain circuits, but the specific sites of adaptation and the molecular changes driving its expression remain unclear. Here, *in situ* hybridization histochemistry was used to examine regulation of select stress-associated signaling systems in brain regions representing likely candidates to underlie exercise-enhanced stress habituation. Analyzed brains were collected from active (6 weeks of wheel running) and sedentary rats following control, acute, or repeated noise exposures that induced a significantly faster rate of glucocorticoid response habituation in active animals but preserved acute noise responsiveness. Nearly identical experimental manipulations also induce a faster rate of cardiovascular response habituation in exercised, repeatedly stressed rats. The observed regulation of the corticotropin-releasing factor and brain-derived neurotrophic factor systems across several brain regions suggests widespread effects of voluntary exercise on central functions and related adaptations to stress across multiple response modalities.

## Introduction

Stress is a significant risk factor for numerous physical (Brindley and Rolland, 1989; Khansari et al., 1990; Forsen, 1991; Pasternac and Talajic, 1991; Vanitallie, 2002; Kalantaridou et al., 2004) and psychological (Dunner et al., 1979; Brown et al., 1987; Hammen et al., 1992; Arborelius et al., 1999; Vanitallie, 2002; Swaab et al., 2005) disorders. Remarkably, many of the same disorders precipitated or exacerbated by stress can be prevented or improved by regular physical exercise (Manson et al., 1992; Paffenbarger et al., 1992; Chodzko-Zajko and Moore, 1994; Bérard et al., 1997; Wannamethee et al., 1998; Dunn et al., 2001; Goodwin, 2003), suggesting that routine physical activity may benefit health in part through a stress-mitigating effect (Roth and Holmes, 1985; Brown and Lawton, 1986; Moraska and Fleshner, 2001; Fleshner, 2005). Indeed, exercise can reduce some of the physiological consequences of acute stress under certain experimental conditions (Brown and Siegel, 1988; Dishman et al., 1995, 1997; Moraska and Fleshner, 2001; Greenwood et al., 2003a,b, 2007a,b; Adlard and Cotman, 2004; Campeau et al., 2010; Masini et al., 2011). However, results are inconsistent across stress stimuli when hypothalamic-pituitary-adrenocortical (HPA) axis-mediated glucocorticoid responses are assessed (Dishman et al., 1995, 1997; Fleshner, 2000; Campisi and Fleshner, 2003; Droste et al., 2003, 2006, 2007; Fediuc et al., 2006; Sasse et al., 2008; Nyhuis et al., 2010), and only limited data are available with regard to cardiovascular reactivity (Morimoto et al., 2000; Masini et al., 2011). Collectively, these findings argue against generalized reductions in acute stress sensitivity by prior exercise, and rather point to complex regulation involving selective modulation of responses to some, but not all, acute stress stimuli. How such a high degree of regulatory specificity is conferred by regular exercise remains to be understood.

While many stress-responsive systems regulate vital physiological functions under both normal and acute stress conditions, it is their sustained, excessive or dysregulated activation by repeated stress that is most strongly associated with pathogenesis (Chrousos and Gold, 1992; Tsigos and Chrousos, 1994; Charmandari et al., 2005). Repeated exposure to the same stressor is often accompanied by a progressive decrease in response amplitude, or habituation, which is considered an adaptation that lessens the physiological toll of repeatedly activating stress-responsive systems (Armario et al., 1984, 1986; Kant et al., 1985; Melia et al., 1994; Campeau et al., 2002). Dysfunctional response habituation could contribute to increased susceptibility to stress-related pathology. Prior work in our laboratory indicated that habituation of glucocorticoid (Sasse et al., 2008; Nyhuis et al., 2010) and tachycardia (Masini et al., 2011) responses to repeated audiogenic stress is facilitated after chronic voluntary wheel running in rats, lending support to the hypothesis that the health benefits of regular exercise include enhanced adaptive mechanisms that reduce the cumulative impact of repeated or prolonged stress. Habituation of stress responses involves active plastic processes mediated by changes within the central nervous system (Armario et al., 1988; Melia et al., 1994; Akana and Dallman, 1997; Ezzeddine and Glanzman, 2003; Esdin et al., 2010). Enhanced glucocorticoid and cardiovascular response habituation to repeated stress in exercised animals is thus likely to result from regulation at a central level by physical activity. To explore this possibility, *in situ* hybridization histochemistry was performed on brains collected from animals that displayed exercise-induced facilitation of glucocorticoid response habituation to repeated audiogenic stress (Sasse et al., 2008; Experiment 2). Similar experimental manipulations also produce enhanced cardiovascular response habituation in exercised, repeatedly stressed rats (Masini et al., 2011). The primary goal was to define neuroanatomical regions and associated neurochemical substrates potentially underlying facilitation of response habituation to repeated audiogenic stress by prior exercise. Although correlational, any reliable changes in central gene expression could subsequently be tested for their specific relevance to the regulation of stress adaptation by physical activity.

First, messenger RNA (mRNA) expression of the immediate-early gene FBJ osteosarcoma oncogene (*Fos*, also known as c-*fos*) was characterized in select regions based on their prior association with audiogenic stress activation. These included the central control station of HPA axis activation, the paraventricular nucleus of the hypothalamus (PVN), in addition to various cortical (cingulate [CG], infralimbic [IL], orbitofrontal/claustrum [OFC/CL], and prelimbic [PL] regions of the medial prefrontal cortex [mPFC]) and forebrain (septohypothalamic nucleus [SHy] and closely associated ventrolateral septum [LSv], anterior bed nucleus of the stria terminalis [BNST]) areas (Campeau and Watson, 1997; Campeau et al., 2002; Burow et al., 2005). Regions differentially activated by repeated audiogenic stress in exercised vs. sedentary brains could reflect important sites of exercise-induced regulation associated with stress response habituation. Within many of these regions, additional molecules reported to mediate glucocorticoid or cardiovascular responses to stress and/or to be regulated by stress or regular exercise were also examined for putative transcriptional regulation. These included transcripts of corticotropin-releasing hormone (*Crh*) and arginine vasopression (*Avp*) at the level of the PVN (Timofeeva et al., 2003; Kawashima et al., 2004; Park et al., 2005), *Crh* in stress-related extra-hypothalamic regions (e.g., BNST, central [CeA] and basolateral [BLA] amygdaloid nuclei—Antoni, 1993; Gu et al., 2003; Dong and Swanson, 2006; Hauger et al., 2006; Radley et al., 2009; Ulrich-Lai and Herman, 2009), *Crh* receptor subtypes *Crhr1* and *Crhr2* (LSv, PVN, BLA and medial amgydaloid nucleus [MeA], and ventromedial hypothalamic nucleus [VMH]—Luo et al., 1994; Chalmers et al., 1995; Makino et al., 1995; Imaki et al., 1996; Van Pett et al., 2000), brain-derived neurotrophic factor (*Bdnf*; hippocampus, PVN—Smith et al., 1995a,b; Neeper et al., 1996; Oliff et al., 1998; Nibuya et al., 1999; Adlard and Cotman, 2004; Farmer et al., 2004; Gomez-Pinella et al., 2008; Nyhuis et al., 2010), and tyrosine receptor kinase B subtype (*Trkb*), the main receptor for *Bdnf*, in the hippocampus (Nibuya et al., 1999). Our group previously examined neurotrophin regulation by prior exercise and repeated stress (Nyhuis et al., 2010), but this analysis did not include comparisons in non-stressed and acutely stressed animals, which were available in the current study.

## Materials and methods

### Subjects and experimental design

Readers are referred to Sasse et al. (2008) (Experiment 2) for explicit details regarding manipulation of animal subjects and experimental design. All animal procedures were reviewed and approved by the Institutional Animal Care and Use Committee of the University of Colorado and conformed to the National Research Council's *Guide for the Care and Use of Laboratory Animals* (8th Edn., 2011). All efforts were made to minimize animal suffering and the number of animals used. Briefly, following an acclimation period to our colony facility, the 48 young adult (~2 months of age upon arrival), male Sprague–Dawley rats (Harlan, Indianapolis, IN) were divided into six groups matched for body weight (*n* = 8/group) and individually housed. Three groups were assigned to the Exercise (Ex) condition and given 24 h unlimited access to a stainless steel running wheel (Nalge Nunc International) attached to the wire lid of their home cages for the 6 weeks prior to and the 11 days of the experimental testing phase. During the same time period, the remaining 3 groups were individually housed in similar cages in the same colony room, but under Sedentary (Sed) conditions, without running wheels in their home cages.

**Figure 1:**
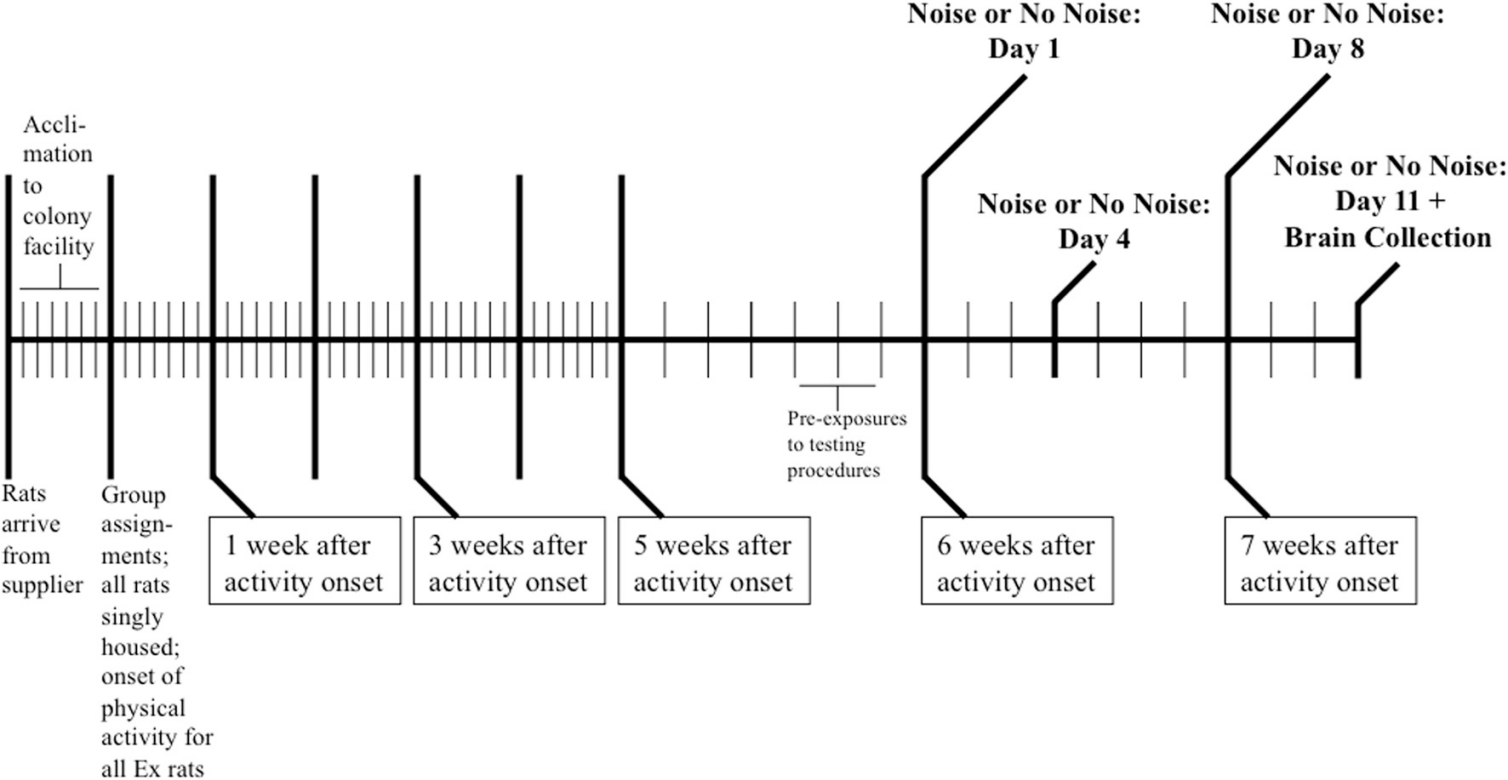
**Schematic depicting the experimental design used in the** Sasse et al. (2008) **study to generate the brain tissue samples utilized in the present study**. Following a 7-day acclimation period to the colony facility, rats were assigned to one of six groups matched for body weight (*n* = 8/group) and individually housed. Half of the rats were given unrestricted voluntary access to running wheels in their home cages (Ex) while the remaining animals lived in similar cages without running wheels under sedentary conditions (Sed) for 6 weeks. Rats were then exposed to 11 consecutive daily 30 min 98 dB noise stress (Ex- or Sed-Repeated Noise; *n* = 8/group) or 60 dB background noise (Ex- or Sed-No Noise; *n* = 8/group) presentations, or were presented with 10 consecutive daily 30 min exposures to 60 dB background noise followed by a single 30 min 98 dB noise stress exposure on Day 11 (Ex- or Sed-Acute Noise; *n* = 8/group). On each of the 3 days prior to the onset of testing, all rats were pre-exposed to the acoustic chambers in order to familiarize them with the testing conditions. Immediately following the stress (or no stress) exposure on Day 11, animals were decapitated and brains were collected for analysis by *in situ* hybridization histochemistry. Each vertical line in the schematic represents 1 day.

Figure 1 provides a graphic summary of the experimental design. On each of the 3 days prior to the onset of testing, all Ex and Sed animals were pre-exposed to the testing conditions, which involved transporting rats to a separate testing room, placing them within their home cages into assigned acoustic chambers, and exposing them only to the background noise (~60 decibel-A scale [dB]) generated by the ventilation fans for 30 min. Importantly, Ex rats would be denied running wheel access during all 30 min stress (or no stress) exposures, so they were habituated to this additional experimental manipulation during these pre-exposures by placing a long metal rod through both ends of each wheel that completely blocked rotation. Then, on each of 11 consecutive days, rats were transported to the testing room, placed within their home cages into their assigned acoustic chambers, and exposed to one of the following *Stress Treatment* conditions for 30 min: 98 dB noise stress (Ex- or Sed-Repeated Noise, *n* = 8/group), 60 dB background noise (Ex- or Sed-No Noise, *n* = 8/group), or 60 dB background noise exposures on the first 10 days followed by a single 98 dB noise stress presentation on Day 11 (Ex- or Sed-Acute Noise, *n* = 8/group). Running wheels of all Ex rats were locked during each noise (or no noise) exposure, after which wheels were immediately unlocked and all animals were returned to the colony. Immediately following the final 30 min 98 dB or background noise exposure on Day 11, all rats were killed by decapitation (without anesthesia), and brains were removed immediately and frozen in isopentane chilled to −30 to −40°C prior to storage at −80°C until further processing.

### *in situ* hybridization histochemistry

Ten micron coronal sections of collected brains were cut on a cryostat (Leica model 1850, Wetzlar, Germany), thaw-mounted onto poly-L-lysine-coated slides, and stored at −80°C until processed. Slides were first fixed in a phosphate-buffered 4% paraformaldehyde solution for 1 h, and then rinsed 3 times in 2X sodium saline citrate (SSC), acetylated for 10 min in 0.1 M triethanolamine containing 0.25% acetic anhydride, rinsed in distilled water, and dehydrated in graded ethyl alcohol concentrations. ^35^S-labeled cRNA riboprobes were generated for *Fos*, *Crh*, *Crhr1*, *Crhr2*, *Avp*, *Bdnf*, and *Trkb* from cDNA subclones in transcription vectors using standard *in vitro* transcription methodology. The rat *Fos* (courtesy of Dr. T. Curran, St. Jude Children's Research Hospital, Memphis, TN) and *Crh* (courtesy of Dr. R. T. Thompson, University of Michigan) cDNA clones were subcloned in pGem 3Z and cut with HindIII to yield 680 nucleotide (nt) and 770 nt cDNA templates, respectively. The rat *Crhr1* cDNA clone (Dr. J. P. Herman, University of Cincinnati) was subcloned in pCR-Blunt II-Topo and cut with BamHI to yield a 345 nt cDNA template. The rat *Crhr2* (courtesy of Dr. R. T. Thompson, University of Michigan) and *Trkb* (courtesy of Dr. D. McKinnon, SUNY, Stony Brook) cDNA clones were subcloned in pBluescript SK and cut with HindIII to yield 899 nt and 306 nt cDNA templates, respectively. The rat *Avp* cDNA clone (courtesy of Dr. T. G. Sherman, Georgetown University Medical Center) was subcloned into pGem 3 and cut with EcoRI to yield a 235 nt cDNA template. The rat *Bdnf* cDNA clone (courtesy of Dr. J. P. Herman, University of Cincinnati) was subcloned in pBluescript and cut with PvuII to yield a 759 nt cDNA template.

Copy riboprobes were labeled in a reaction mixture consisting of 1 μ g of the appropriate linearized plasmid, 1X T3, T7, or SP6 transcription buffer (Promega), 125 μ Ci ^35^S-UTP, 150 μ M NTP's (ATP, CTP, and GTP), 12.5 mM dithiothreitol, 20 U RNase inhibitor, and 6 U of T3 (for *Crhr2*, *Bdnf*, and *Trkb*), T7 (for *Fos*, *Crh*, and *Crhr1*), or SP6 (for *Avp*) RNA polymerase. The reaction was allowed to proceed for 2 h at 37°C, after which probe was separated from free nucleotides over a Sephadex G50–50 column. Riboprobes were diluted in hybridization buffer consisting of 50% formamide, 10% dextran sulfate, 2X SSC, 50 mM sodium phosphate buffer (pH 7.4), 1X Denhardt's solution, and 0.1 mg/ml yeast tRNA, to yield ~1–2 × 10^6^ dpm/70 μ l buffer. Diluted probe (70 μ l) was applied to tissue sections on each slide, which were then coverslipped, placed in sealed plastic boxes lined with filter paper moistened with 60% formamide in distilled water, and incubated overnight (12–18 h) at 55°C. Coverslips were then removed, and slides were rinsed 3 times in 2X SSC before being incubated in a solution containing RNase A (2.0 μg/ml) for 1 h at 37°C. Slides were next washed successively in 2X, 1X, 0.5X, and 0.1X SSC for 5 min each, and then incubated in 0.1X SSC for 1 h at 65°C. They were subsequently returned to room temperature in 2 rinses of 0.1X SSC for 5–10 min each prior to being dehydrated in graded ethyl alcohols and air-dried. Slides were then exposed to Kodak MR X-ray film for optimized exposure times.

Control experiments were performed on tissue sections pre-treated with RNase A (2.0 μg/ml at 37°C for 1 h) prior to hybridization; this treatment prevented labeling. Additionally, some control sections were hybridized with the sense cRNA strands, which in all cases did not lead to significant hybridization to tissue sections (data not shown). For each riboprobe, two to three slides (4–6 sections/slide) representing a given brain region from each rat included in the study were processed simultaneously to allow for direct comparisons in the same regions. In more cases than not, multiple *in situ* hybridizations were performed for the same target mRNA at different levels of the brain with all animals represented, which reduced the effects of technical variation within regions, but limited the ability to make direct comparisons between different regions.

### Image analysis

Semi-quantitative densitometric analyses were performed on digitized images from X-ray films in the linear range of the gray values obtained with our acquisition system (Northern Light lightbox model B95 [Imaging Res. Inc., St. Catharines, Ontario], a SONY TV camera model XC-ST70 fitted with a Navitar 7000 zoom lens [Rochester, NY], connected to an LG3-01 frame grabber [Scion Corp., Frederick, MD] inside a Dell Dimension 500, captured with Scion Image beta rel. 4.02). Signal pixels in a region of interest were defined as being 3.5 standard deviations above the mean gray value of the representative background, which was set by a cell- and/or signal- poor area close to the region of interest. Mean integrated gray values were computed from the product of the number of pixels comprising the positive signal and their average gray level within the region of interest.

**Figure 2:**
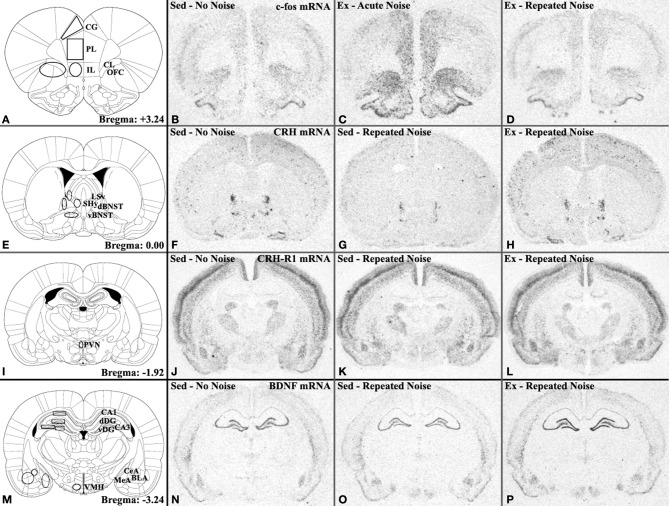
**Diagram specifying brain regions analyzed by *in situ* hybridization and corresponding representative autoradiograms depicting important groups differences.** Panels in the left column provide images adapted from the Paxinos and Watson (2005) atlas (with permission to reprint from Elsevier) of coronal brain sections at the level of the **(A)** medial prefrontal cortex (mPFC), **(E)** ventrolateral septum (LSv) and anterior bed nucleus of the stria terminalis (BNST), **(I)** paraventricular nucleus of the hypothalamus (PVN), and **(M)** hippocampus and amygdala. All regions of interest analyzed in this study are labeled on the right sides of these sections while the left sides depict the templates used to consistently define their boundaries. Remaining panels in each row show representative autoradiogram images of specified mRNAs in brain sections corresponding to these atlas diagrams. Panels **(B–D)** *Fos* mRNA expression illustrating robust induction of *Fos* mRNA by acute noise stress and nearly complete habituation of *Fos* responses to levels similar to No Noise controls following repeated noise exposures in some (prelimbic [PL] and infralimbic [IL]), but not all (orbitofrontal cortex/claustrum [OFC/CL] and cingulate [CG]) mPFC regions. Panels **(F–H)** *Crh* mRNA in the dorsal (d) and ventral (v) aspects of the BNST demonstrating repeated noise-induced reduction of *Crh* mRNA expression in sedentary but not exercised rats, which exhibited levels similar to No Noise controls. Panels **(J–L)** *Crhr1* mRNA expression in the PVN illustrating the trend for mRNA induction that was specific to exercised, repeatedly stressed rats. Panels **(N–P)** *Bdnf* mRNA expression showing the trend for voluntary exercise to prevent the repeated stress-induced reduction in hippocampal *Bdnf* mRNA expression observed in sedentary rats. Other abbreviations: BLA, basolateral nucleus of amygdala; CeA, central nucleus of amygdala; dDG, dorsal dentate gyrus; MeA, medial nucleus of amygdala; SHy, septohypothalamic nucleus; vDG, ventral dentate gyrus; VMH, ventromedial nucleus of hypothalamus.

The panels in the left column of Figures 2A,E,I,M provide schematic representations of the brain that were adapted from *The Rat Brain in Stereotaxic Coordinates* (CD-ROM; Paxinos and Watson, 2005) atlas, with permission from Elsevier. The right sides of these coronal sections are labeled for the regions of interest in which semi-quantitative analyses were performed, while the left sides of the same sections illustrate the templates that were consistently used to define these regions. For example, panel **A** represents a section at the level of the prefrontal cortex (PFC; 3.24 mm anterior to bregma, as designated in the lower right corner), in which *Fos* mRNA expression was analyzed in: the prelimbic (PL) PFC, designated by a 50 × 60 pixel rectangle template; IL PFC, designated by a 40 × 50 pixel ellipse; the region encompassing the orbitofrontal cortex and claustrum (OFC/CL), designated by a 110 × 60 pixel ellipse; and the cingulate (CG) PFC, for which a trapezoidal template was designed using the Paxinos and Watson atlas for guidance. For each riboprobe, 4–12 bilateral measurements were made within each relevant region of interest (as defined by the template boundaries illustrated in Figure 2) on appropriate sections from each animal. These values were averaged to obtain the mean integrated gray value per region for each rat, which provided the relative mRNA expression level. This analytic method gives relative semi-quantitative results that are comparable to performing a quantitative grain analysis on photographic emulsion-dipped sections (Day et al., 2005). Occasionally, sections or slides for a specific animal were missing, damaged, or otherwise inappropriate for analysis following these multiple levels of processing and were therefore excluded. These instances will be explicitly stated in the appropriate Results section.

### Statistical analyses

All statistical analyses were performed using the *Statistical Analysis Software (SAS)* program. Two-way (*Activity Status* × *Stress Treatment*) ANOVAs were used to analyze the various target mRNA expression levels in the brain regions of interest. These were followed by Scheffé's *post-hoc* multiple means comparisons to determine the source of reliable effects when present. Statistical significance for all analyses was set at *p* ≤ 0.05.

## Results

### Regional Fos mRNA induction

Expression levels and basic statistical comparisons of *Fos* mRNA measured in several brain regions of exercised and sedentary rats immediately following the last of 11 consecutive daily noise (Repeated Noise) or background noise (No Noise) exposures, or an acute noise stress presentation on Day 11 (Acute Noise), are presented in Table 1. Of the eight regions analyzed, six displayed a similar pattern of *Fos* mRNA induction, in which only rats from the acutely exposed groups demonstrated reliable induction that was greater than that of the repeatedly exposed and no noise control groups, which did not differ from each other (see panels **B–D** of Figure 2). These regions included the PVN [main effect of *Stress Treatment*: *F*_(2, 38)_ = 35.73, *p* < 0.0001], LSv [*Stress Treatment*: *F*_(2, 42)_ = 55.77, *p* < 0.0001], SHy [*Stress Treatment*: *F*_(2, 42)_ = 82.27, *p* < 0.0001], ventrolateral aspect of the anterior BNST [vBNST; *Stress Treatment*: *F*_(2, 42)_ = 23.64, *p* < 0.0001], and the PL [*Stress Treatment*: *F*_(2, 38)_ = 6.93, *p* = 0.0027] and IL [*Stress Treatment*: *F*_(2, 38)_ = 17.15, *p* < 0.0001] regions of the mPFC, as evidenced by Two-Way ANOVAs and confirmed and further characterized by Scheffé's *post-hoc* analyses (all *p*'s ≤ 0.05). Additional Two-Way ANOVAs showed that group differences in *Fos* mRNA expression in the OFC/CL region did not reach statistical significance, and while there was a significant *Activity Status* × *Stress Treatment* interaction effect on *Fos* mRNA expression in the CG [*F*_(2, 38)_ = 3.28, *p* = 0.0487], Scheffé's *post-hoc* analyses did not reveal any reliable differences among pair-wise comparisons. Of the four mPFC regions examined for *Fos* mRNA induction, the following groups had at least one animal excluded from the analysis: Sed-Repeated Noise (*n* = 7), Ex-No Noise (*n* = 7), and Ex-Repeated Noise (*n* = 6). The following groups had at least one animal excluded from the analysis in the PVN: Sed-Repeated Noise (*n* = 7) and Ex-Repeated Noise (*n* = 7).

**Table 1:** **Stress and voluntary exercise effects on central *Fos* mRNA expression**.

	**Sed**			**Ex**		
	**No**	**Acute**	**Repeated**	**No**	**Acute**	**Repeated**
**Fos mRNA**						
**Cortex**						
CG	141 (50)	243 (58)	194 (41)	288 (93)	163 (23)	94 (17)
IL^*^^†^	19 (5.4)^a^	101 (18)^b^	37 (9.5)^a^	38 (13)^a^	78 (9.6)^b^	34 (7.2)^a^
OFC/CL	369 (86)	568 (105)	577 (159)	554 (146)	412 (145)	339 (51)
PL^*^^†^	105 (38)^a^	333 (67)^b^	184 (60)^a^	182 (52)^a^	281 (36)^b^	132 (24)^a^
**Forebrain**						
vBNST^*^^†^	4.3 (1.2)^a^	51 (11)^b^	5.6 (1.0)^a^	6.0 (2.3)^a^	43 (11)^b^	12 (5.3)^a^
SHy^*^^†^	2.3 (1.2)^a^	178 (18)^b^	16 (5.8)^a^	3.8 (1.5)^a^	144 (27)^b^	18 (6.3)^a^
LSv^*^^†^	6.7 (2.6)^a^	248 (35)^b^	40 (22)^a^	20 (6.4)^a^	225 (33)^b^	57 (16)^a^
**Hypothalamus**						
PVN^*^^†^	6.4 (1.3)^a^	61 (7.8)^b^	10 (2.1)^a^	5.7 (0.9)^a^	48 (13)^b^	8.4 (2.3)^a^

Mean relative Fos mRNA expression in indicated brain regions of exercised (Ex) and sedentary (Sed) rats exposed to no, acute, or repeated noise stress. Group means are mean integrated gray values/100 (±1 s.e.m.), presented as arbitrary units. CG, cingulate cortex; IL, infralimbic cortex; OFC/CL, orbitofrontal cortex/claustrum; PL, prelimbic cortex; vBNST, ventrolateral bed nucleus of stria terminalis; SHy, septohypothalamic nucleus; LSv,ventrolateral septum; PVN, paraventricular nucleus of hypothalamus.

* Omnibus ANOVA, p ≤ 0.05.

† Two-Way ANOVA: significant main effect of Stress Treatment (p ≤ 0.05);

a,b across each row, Stress Treatment group means with same (or no) letter symbol were not statistically different (Scheffé, p ≤ 0.05).

### Hypothalamic Crh and Avp mRNA expression

**Figure 3:**
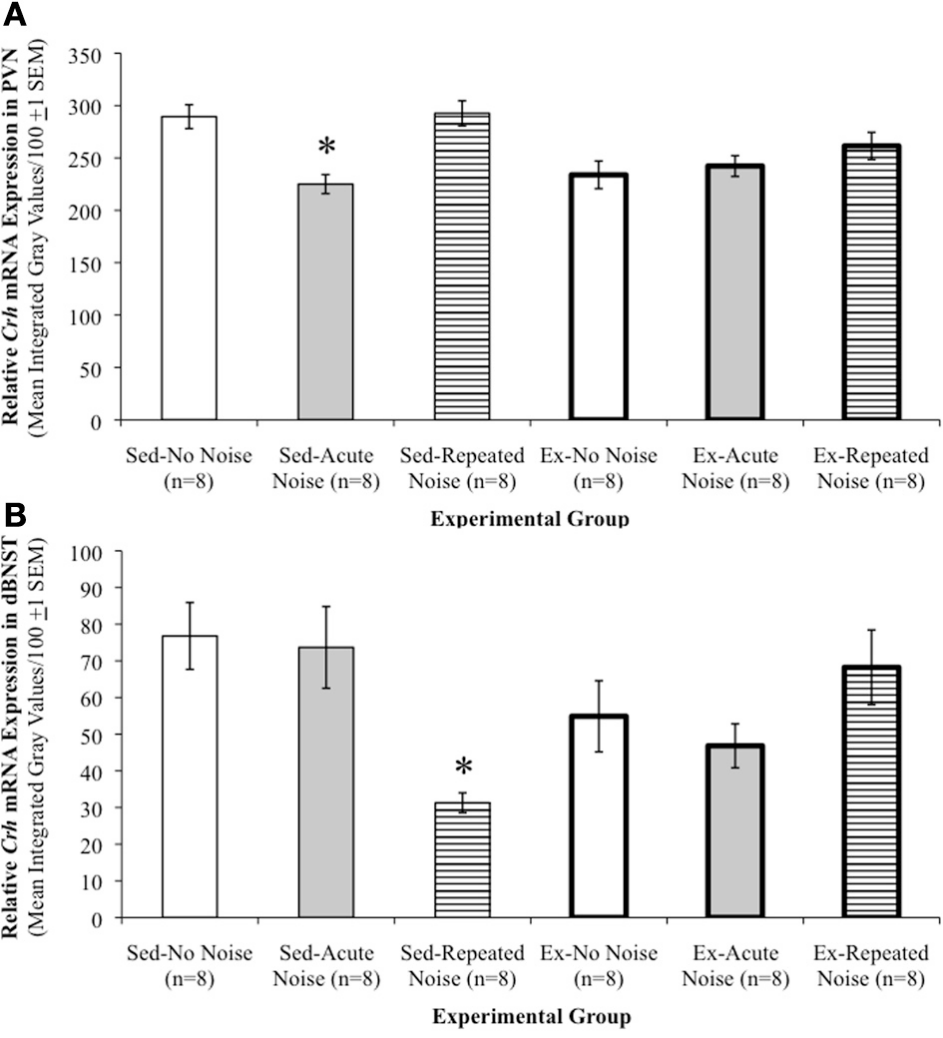
**Stress-induced *Crh* regulation in the PVN and dBNST is different in exercised compared to sedentary rats. (A)** Relative *Crh* mRNA expression levels in the paraventricular nucleus of the hypothalamus (PVN). Significant main effects of *Activity Status* and *Stress Treatment* were observed, with reliably lower PVN-*Crh* mRNA expression levels in Ex as compared to Sed rats, and significantly greater expression levels in Repeated as compared to Acute Noise groups, although neither of these two groups differed reliably from the No Noise controls. The interaction effect was also significant, such that, while acute and repeated noise stress exposure had little effect on *Crh* mRNA expression in the PVN of Ex rats, the Sed rats acutely exposed to noise showed a reliable reduction in mRNA expression as compared to the other two Sed groups, which did not differ. Group means are mean integrated gray values/100 (±1 s.e.m.), presented as arbitrary units. ^*^p ≤ 0.05 vs. Sed-No Noise and Sed-Repeated Noise. **(B)** Relative *Crh* mRNA expression levels in the dorsal aspect of the anterior bed nucleus of the stria terminalis (dBNST). A significant *Activity Status* × *Stress Treatment* interaction effect was found such that, while expression levels were decreased by the repeated noise stress in Sed rats, they were relatively unaffected, and even slightly increased, in Ex animals exposed to the same repeated stress paradigm. A similar pattern in CRH mRNA expression was also observed in the anteroventral portion of the BNST (vBNST), as well as the central nucleus of the amygdala (CeA; see Table 2). Group means are mean integrated gray values/100 (±1 S.E.M.), presented as arbitrary units. ^*^p ≤ 0.05 vs. all other experimental groups, which were not reliably different amongst each other.

*Crh* mRNA expression levels in the PVN are presented in Table 2. A Two-Way ANOVA revealed significant *Activity Status* [*F*_(1, 42)_ = 6.07, *p* = 0.0180], *Stress Treatment* [*F*_(2, 42)_ = 7.28, *p* = 0.0019], and *Activity Status* × *Stress Treatment* [*F*_(2, 42)_ = 5.17, *p* = 0.0098] effects on *Crh* mRNA expression in this key locus of stress response integration. Specifically, when controlling for *Stress Treatment*, Ex rats exhibited reliably lower PVN-*Crh* mRNA expression than Sed rats, and while holding *Activity Status* constant, animals in the Repeated Noise groups had significantly greater mRNA expression than those exposed to Acute Noise, although neither of these two groups differed reliably from the No Noise controls (Scheffé, *p*'s ≤ 0.05). The significant interaction effect reflects that, while acute and repeated noise stress exposure had little effect on *Crh* mRNA expression in the PVN of Ex rats, the Sed rats acutely exposed to noise showed a reliable reduction in mRNA expression as compared to the other two Sed groups, which did not differ (Scheffé, *p* ≤ 0.05). A graphic representation of *Crh* mRNA expression in the PVN is depicted in Figure 3A to help illustrate this interaction. *Avp* mRNA expression was abundant in both the paraventricular and supraoptic nuclei of the hypothalamus in all groups. However, the extent of this expression was not reliably affected by *Activity Status* or *Stress Treatment* in either structure, as evidenced by non-significant Two-Way ANOVAs (data not shown).

**Table 2:** **Effects of stress and wheel running on central CRH signaling**.

	**Sed**			**Ex**		
	**No**	**Acute**	**Repeated**	**No**	**Acute**	**Repeated**
**Crh mRNA**						
**Forebrain**						
dBNST^*^^¶^	77 (9.1)	74 (11)	31 (2.7)	55 (9.7)	47 (6.0)	68 (10)
vBNST^*^^¶^	56 (7.5)	43 (5.2)	28 (6.9)	34 (3.8)	32 (3.7)	52 (6.4)
**Hypothal**.						
PVN^*^^‡^^†^^¶^	289 (11)^a^,^b^	225 (9.1)^b^	293 (12)^a^	234 (13)^a^,^b^	242 (10)^b^	261 (13)^a^
**Amygdala**						
CeA^*^^¶^	136 (16)	137 (24)	73 (10)	93 (16)	67 (15)	119 (18)
**Crhr1 mRNA**						
**Hypothal**.						
PVN	31 (4.5)	33 (8.1)	33 (4.5)	28 (3.5)	24 (4.0)	47 (8.3)
**Amygdala**						
MeA	191 (19)	159 (17)	173 (20)	191 (22)	152 (30)	156 (12)
BLA	148 (19)	99 (8.5)	147 (14)	132 (19)	110 (27)	116 (12)
**Crhr2 mRNA**						
**Forebrain**						
LSv	673 (83)	636 (106)	490 (111)	445 (65)	585 (122)	775 (129)
**Hypothal**.						
VMH^*^^‡^	329 (19)	322 (29)	309 (20)	250 (9.9)	284 (16)	258 (14)
**Amygdala**						
MeA	369 (39)	344 (42)	332 (32)	358 (24)	378 (36)	335 (48)

Mean relative expression of Crh, Crhr1, and Crhr2 mRNAs in indicated brain regions of Ex and Sed rats following no, acute, or repeated audiogenic stress. Group means are mean integrated gray values/100 (±1 s.e.m.), presented as arbitrary units. dBNST, dorsal BNST; Hypothal., hypothalamus; CeA, central nucleus of amygdala; MeA and BLA, medial and basolateral nuclei of amygdala; VMH, ventromedial nucleus of hypothalamus.

* Omnibus ANOVA, p ≤ 0.05.

Two-Way ANOVA:

‡ *significant main effect of Activity Status*,

† significant main effect of Stress Treatment (p ≤ 0.05);

a, b across each row, Stress Treatment group means with same (or no) letter symbol(s) were not statistically different (Scheffé, p ≤ 0.05). ¶ significant Activity Status x Stress Treatment interaction (p ≤ 0.05); group differences are not indicated by letter symbols here but rather presented in the Results section and illustrated in Figure 3.

### Extra-hypothalamic Crh mRNA expression

Table 2 also depicts relative *Crh* mRNA expression levels in specified extra-hypothalamic regions. In the dorsal aspect of the anterior BNST (dBNST), a significant *Activity Status* × *Stress Treatment* interaction effect was found on *Crh* mRNA expression [*F*_(2, 42)_ = 8.42, *p* = 0.0008]. In general, while expression was decreased by the repeated noise stress in Sed rats, it was relatively unaffected, and even somewhat increased, in Ex animals exposed to the same repeated stress paradigm (see panels **F–H** of Figure 2). The only statistically significant group difference revealed by Sheffé's *post-hoc* analyses, however, was that *Crh* mRNA expression was reliably lower in the Sed-Repeated Noise group than all other groups (*p* ≤ 0.05), which did not differ amongst each other. *Crh* mRNA levels in the dBNST are depicted in Figure 3B to aid in the visualization of this interaction. A similar expression pattern was observed in the vBNST and the CeA, and while significant *Activity Status* × *Stress Treatment* interaction effects were uncovered for both regions by Two-Way ANOVAs [vBNST: *F*_(2, 42)_ = 9.27, *p* = 0.0005; CeA: *F*_(2, 41)_ = 6.62, *p* = 0.0032], none of the *post-hoc* pair-wise comparisons revealed reliable group differences (Scheffé, all *p*'s > 0.05). One animal in the Ex-Acute Noise group was excluded from the analysis of *Crh* mRNA expression in the CeA (*n* = 7).

### Crh receptor mRNAs

Two-Way ANOVAs revealed no significant differences in *Crhr1* mRNA levels in the MeA or BLA. Similarly, no reliable effects of *Activity Status* or *Stress Treatment* were found with respect to *Crhr1* mRNA expression in the PVN when analyzing all six experimental groups (see Table 2). Interestingly, when comparing only the Ex groups using a One-Way ANOVA for *Stress Treatment*, a significant main effect was uncovered [*F*_(2, 21)_ = 4.67, *p* = 0.0210], such that *Crhr1* mRNA expression was significantly greater in the PVN of Ex-Repeated Noise as compared to Ex-Acute Noise animals (Scheffé, *p* ≤ 0.05), although neither of these groups differed reliably from the Ex-No Noise group (see panels **J–L** of Figure 2). A Two-Way ANOVA revealed a significant main effect of *Activity Status* [*F*_(1, 40)_ = 13.88, *p* = 0.0006] on *Crhr2* mRNA expression in the VMH, with reliably lower expression in brains of Ex compared to Sed animals. The following groups had at least one animal excluded from the analysis in the VMH: Sed-Acute Noise (*n* = 7) and Sed-Repeated Noise (*n* = 7). *Crhr2* mRNA levels were not reliably different between treatment groups in the LSv or MeA (Table 2).

### Bdnf and Trkb mRNA expression

**Figure 4:**
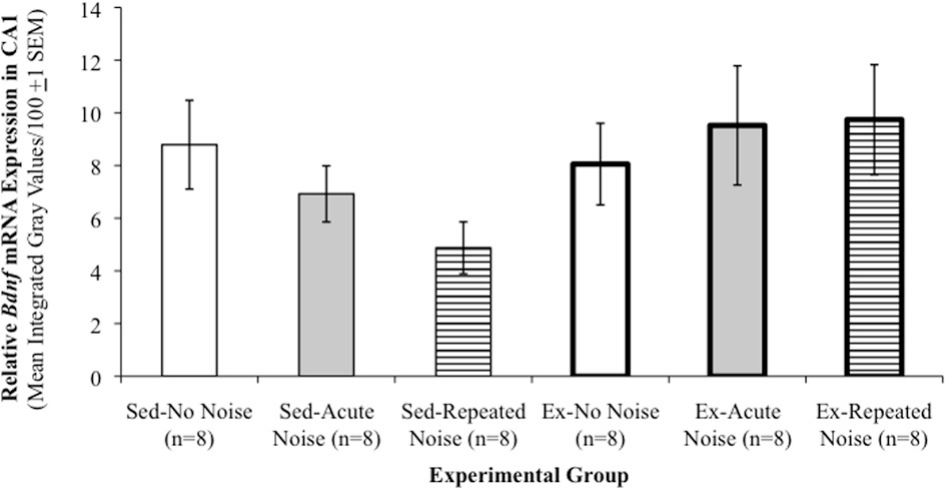
**Exercised rats appear less susceptible to stress-induced decreases in hippocampal *Bdnf*.** Relative *Bdnf* mRNA expression levels in the CA1 sub-region of the hippocampus. Although group differences did not achieve statistical significance, a general pattern in *Bdnf* mRNA expression clearly emerged in this and the other three hippocampal sub-regions examined that is well-demonstrated by this particular figure, in that Ex rats did not appear to be susceptible to the same acute or repeated stress-induced decreases in hippocampal *Bdnf* mRNA expression that were exhibited in the Sed animals. Group means are mean integrated gray values/100 (±1 s.e.m.), presented as arbitrary units.

Expression levels and basic statistical comparisons of *Bdnf* and *Trkb* transcripts are presented in Table 3. In three of the four hippocampal sub-regions examined for *Bdnf* mRNA expression, Ex rats displayed significantly greater expression than Sed animals when controlling for *Stress Treatment*, as shown by Two-Way (*Activity Status* × *Stress Treatment)* ANOVAs. These included the CA3 region [main effect of *Activity Status*: *F*_(1, 42)_ = 17.22, *p* = 0.0002] and the dorsal [*Activity Status*: *F*_(1, 40)_ = 33.04, *p* < 0.0001] and ventral [*Activity Status*: *F*_(1, 42)_ = 27.70, *p* < 0.0001] blades of the dentate gyrus. Relatively low levels of *Bdnf* mRNA were expressed in the CA1 region that did not differ reliably between the experimental groups. Although not statistically significant, a general pattern in hippocampal *Bdnf* mRNA expression emerged such that, in Sed rats, acute and repeated noise had a tendency to decrease expression relative to non-stressed controls, while Ex animals appeared to be buffered from this stress-induced reduction in hippocampal *Bdnf* transcription (see panels **N–P** of Figure 2). Figure 4 depicts *Bdnf* mRNA expression levels in the CA1 region, which serves to clearly illustrate this pattern of results. Expression of *Bdnf* mRNA was also examined in the PVN, in which a Two-Way ANOVA uncovered no reliable group differences. A significant main effect of *Activity Status* was revealed by Two-Way ANOVAs on *Trkb* mRNA expression in the CA1 [*F*_(1, 42)_ = 8.63, *p* = 0.0053] and CA3 [*F*_(1, 42)_ = 5.37, *p* = 0.0254] hippocampal sub-regions, such that mRNA levels were significantly greater in Ex compared to Sed rats (Scheffé, *p's ≤ 0.05)*. No reliable group differences in *Trkb* mRNA expression were observed in either the dorsal or ventral blades of the dentate gyrus.

**Table 3:** **Stress and wheel running regulation of central *Bdnf* and *Trkb* mRNA expression**.

	**Sed**			**Ex**		
	**No**	**Acute**	**Repeated**	**No**	**Acute**	**Repeated**
***Bdnf* mRNA**						
**Hippocampus**						
CA1	8.8 (1.7)	6.9 (1.1)	4.9 (0.9)	8.1 (1.5)	9.5 (2.3)	9.7 (2.1)
CA3^*^^‡^	100 (8.0)	91 (4.9)	82 (8.4)	114 (8.4)	124 (12)	124 (9.2)
dDG^*^^‡^	138 (7.4)	125 (7.4)	115 (16)	169 (14)	187 (14)	189 (12)
vDG^*^^‡^	113 (10)	95 (5.9)	93 (14)	148 (17)	170 (19)	163 (15)
**Hypothalamus**						
PVN	111 (15)	113 (13)	117 (11)	100 (16)	118 (16)	126 (16)
***Trkb* mRNA**						
**Hippocampus**						
CA1^*^^‡^	106 (7.2)	96 (4.7)	100 (7.3)	121 (8.2)	118 (4.4)	107 (3.0)
CA3^‡^	82 (7.9)	72 (7.5)	80 (6.6)	97 (10)	95 (9.0)	87 (5.8)
dDG	133 (9.2)	121 (8.0)	129 (6.7)	139 (9.5)	144 (11)	136 (3.7)
vDG	145 (11)	132 (9.9)	143 (8.2)	148 (8.6)	155 (9.5)	136 (4.6)

Mean relative Bdnf and Trkb mRNA expression levels in indicated brain regions of Ex and Sed rats following no, acute, or repeated stress. Group means are mean integrated gray values/100 (±1 s.e.m.), presented as arbitrary units.

dDG and vDG, dorsal and ventral blades of dentate gyrus, respectively.

* Omnibus ANOVA, p ≤ 0.05.

Two-Way ANOVA:

‡ significant main effect of Activity Status (p ≤ 0.05).

## Discussion

The results of this study indicate that physical activity can significantly alter central nervous system expression patterns of several genes strongly associated with stress responsiveness. As summarized below, a number of studies have already identified various genes that are differentially regulated in the brains of physically active compared to sedentary rats under control (no stress) conditions. Importantly, some of this regulation can be further differentiated in exercised and sedentary brains in response to acute or repeated stress exposures. In view of our recent findings that prior physical activity enhances the rate of neuroendocrine and autonomic response habituation to repeated stress (Sasse et al., 2008; Nyhuis et al., 2010; Masini et al., 2011), we reasoned that these previously reported sites and molecular targets of regulation could also underlie this particular mode of stress adaptation. While the brains analyzed in the current study were obtained following measurements of HPA axis activation (Sasse et al., 2008), a nearly identical experimental protocol led to significantly facilitated habituation of heart rate and core body temperature responses in exercised rats (Masini et al., 2011). It is therefore highly likely that the central gene regulation observed in the current study could be associated with modulation of either or both the neuroendocrine and autonomic response adaptations observed in our prior studies.

### Regional *Fos* mRNA induction and habituation

As reported previously (Campeau et al., 1997, 2002; Campeau and Watson, 1997; Burow et al., 2005), acute loud noise exposure induced widespread *Fos* mRNA induction, but importantly, the amplitude of this expression did not differ between exercised and sedentary rats. This finding supports a number of prior studies reporting that acute HPA axis (Dishman et al., 1995, 1997, 1998; Fleshner, 2000; Fediuc et al., 2006; Sasse et al., 2008; Campeau et al., 2010; Nyhuis et al., 2010) and autonomic (Morimoto et al., 2000; Salam et al., 2009; Masini et al., 2011) responses to stress do not always differ between sedentary and more active animals. These indices of neuronal activation provide further evidence that general central responsiveness to acute stress can be preserved in exercising animals, which is important given the critical role of stress responses in adaptation and survival (Munck et al., 1984; Levine and Ursin, 1991; Chrousos and Gold, 1992; Akil and Morano, 1995). Notably, whereas our previous work demonstrated enhanced HPA axis (Sasse et al., 2008; Nyhuis et al., 2010) and autonomic (Masini et al., 2011) response habituation to repeated stress exposures in exercised animals, the quantified brain regions of Ex and Sed rats did not exhibit significant differences in *Fos* mRNA induction immediately following the final (11th) repeated noise exposure. This general result may reflect the fact that brains were collected at a point during repeated stress exposures at which the neuroendocrine and autonomic response differences between exercised and sedentary rats have mostly dissipated (Sasse et al., 2008; Nyhuis et al., 2010; Masini et al., 2011). Future studies should include earlier time-points during repeated stress exposures (3rd–8th exposures) when HPA axis and autonomic response differences are observed. In addition, although non-significant, an interesting pattern emerged in the brains of exercised animals, such that acute and especially repeated noise stress appeared to *decrease Fos* mRNA induction in prefrontal cortical regions relative to that of No Noise controls (see Table 1). Prior associations with motivated behaviors (Cardinal et al., 2002) and stress adaptation (Campeau et al., 2002; Weinberg et al., 2009) make these regions important targets for future studies of the impact of physical activity on stress adaptation.

### Crh and Avp expression in the paraventricular hypothalamic nucleus

In the PVN, stress-induced release of CRH and AVP peptides are typically accompanied by increases in their gene transcription (Herman et al., 1992; Kovács and Sawchenko, 1996; Ma et al., 1997; Girotti et al., 2006) and subsequent elevations of steady state mRNA levels (Herman et al., 1992; Luo et al., 1994; Makino et al., 1995). It was therefore surprising to observe the lowest PVN-*Crh* mRNA levels in the Sed-Acute Noise group relative to that of the other Sed groups. However, PVN-*Crh* mRNA increases are typically observed 1–4 h after stress onset (Herman et al., 1992; Luo et al., 1994; Ma et al., 1997) and thus may have been missed given the shorter 30 min interval employed in the current study. This may also explain the lack of a *Stress Treatment* effect on the expression of *Avp* mRNA in the PVN, as elevation in *Avp* transcription is further delayed compared to that of *Crh* (Herman et al., 1992; Kovács and Sawchenko, 1996; Ma et al., 1997). An alternative explanation might be provided by unusually elevated *Crh* mRNA expression levels in the Sed-No Noise rats. Importantly, the lack of differences in plasma corticosterone concentrations between exercised and sedentary rats under basal conditions, after acute stress exposure, and following 11 days of repeated stress exposures (Sasse et al., 2008; Nyhuis et al., 2010) suggests that observed differences in PVN-*Crh* mRNA exerted little influence on HPA axis tone or sensitivity. Repeated stress is also reported to elevate *Crh* mRNA expression in the PVN (Aguilera, 1994; Herman et al., 1995; Gómez et al., 1996), yet the comparatively milder repeated noise stress paradigm used here did not produce significant increases in PVN-*Crh* or *Avp* mRNA expression in sedentary or exercised rats. The similar expression levels of these principal hypothalamic regulators of stress responses between exercised and sedentary rats therefore argues against an important role for these hypophysiotropic signals in the differential rates of stress adaptation we have observed (Sasse et al., 2008; Nyhuis et al., 2010; Masini et al., 2011).

### Extra-hypothalamic *Crh* regulation

*Crh* mRNA expression was examined in three additional, extra-hypothalamic regions, including the central amygdala and the dorsal and ventral BNST. Previous studies have demonstrated that both acute and repeated stress activate the CRH systems in these regions (Chappell et al., 1986; Merali et al., 1998; Makino et al., 1999; Figueiredo et al., 2003) and have established important roles for their recruitment in mediating the expression and integration of behavioral and autonomic components of the stress response (Fisher et al., 1982; Koob et al., 1993). Each of these three regions exhibited a similar and interesting pattern of *Crh* regulation, in which expression was generally decreased by repeated noise stress in sedentary rats but was relatively unaffected, if not increased (although non-significantly), in voluntarily exercising animals exposed to the same repeated stress paradigm. The relatively short, 30 min interval between stress onset and euthanasia may again account for the lack of clear acute stress effects in these regions when compared to prior studies (Chappell et al., 1986; Merali et al., 1998; Makino et al., 1999; Figueiredo et al., 2003). Based on the results obtained in the Sed groups, one might hypothesize that a decrease in extra-hypothalamic CRH contributes to response habituation to repeated stress exposures. If this were the case, an even greater reduction in extra-hypothalamic *Crh* transcript would be expected in the Ex-Repeated Noise group, since response habituation to repeated noise was enhanced in these animals relative to their sedentary counterparts (Sasse et al., 2008; Nyhuis et al., 2010; Masini et al., 2011), but this was not observed. Whether and how differential regulation of these extra-hypothalamic influences contributes to the different rates of response habituation exhibited by exercised and sedentary rats remains an open question.

### Regulation of *Crh* receptors

Expression levels of *Crhr1* mRNA in the medial and basolateral amygdala were similar between Ex and Sed groups, and were relatively insensitive to the different *Stress Treatment* conditions of this experiment, which is consistent with previous observations (Van Pett et al., 2000). In the PVN, *Crhr1* mRNA is typically expressed at very low levels basally, although several groups have reported inducible expression of PVN-*Crhr1* following exposure to a variety of different stressors (Luo et al., 1994; Makino et al., 1995; Rivest et al., 1995; Imaki et al., 1996; Van Pett et al., 2000). The only reliable difference in *Crhr1* expression observed in this study was exhibited in the PVN specifically of Ex rats, with significantly greater expression in the Repeated Noise compared to the Acute Noise group (although neither group differed significantly from No Noise controls). While others have demonstrated *Crhr1* mRNA induction in the PVN several hours following stressor onset (e.g., Van Pett et al., 2000), its expression was below the threshold of detection in the PVN of both Ex and Sed rats 30 min after acute noise onset. It thus remains possible that differential regulation of PVN-*Crhr1* might be revealed at later time-points following acute stress exposure.

Exploration of *Crhr2* mRNA expression, which is regionally more limited than that of *Crhr1* (Chalmers et al., 1995; Van Pett et al., 2000), did not reveal any differences in the lateral septum or medial amygdala. However, *Crhr2* mRNA expression was significantly reduced in the ventromedial hypothalamic nucleus of exercised compared to sedentary groups. Since *Crhr2* signaling in this region has previously been implicated in the regulation of food intake and energy balance (Steller, 1954; Spina et al., 1996), and significant alterations in these same measures are consistently reported in voluntarily exercising rats (Tokuyama et al., 1982; Rodnick et al., 1989; Afonso and Eikelboom, 2003), this finding may be important for future studies aimed at unraveling the complex metabolic adaptations associated with regular exercise.

### Effects within the Bdnf-Trkb signaling system

In three of the four hippocampal sub-regions in which *Bdnf* mRNA levels were assessed, expression was significantly greater in Ex compared to Sed rats. This finding is consistent with several other reports of increased hippocampal *Bdnf* expression in voluntarily exercising animals, both at the mRNA (Neeper et al., 1996; Oliff et al., 1998; Farmer et al., 2004; Gomez-Pinella et al., 2008; Nyhuis et al., 2010) and protein levels (Adlard and Cotman, 2004). Further, although stress has been demonstrated to reduce *Bdnf* expression in the hippocampus and PVN (Smith et al., 1995a,b; Nibuya et al., 1999; Adlard and Cotman, 2004), only non-significant reductions were observed here, particularly in the Sed-Repeated Noise rats. Importantly, the general trends we observed in the hippocampus were consistent with previously published results, in that voluntary exercise appeared to prevent the non-significant stress-induced decreases in hippocampal *Bdnf* mRNA expression observed in sedentary animals. Similarly, greater *Trkb* mRNA levels were expressed in the CA1 and CA3 pyramidal cell layers of Ex compared to Sed rats. Taken together, the exercise-induced increases in both ligand (*Bdnf*) and receptor (*Trkb*) transcripts in the present study are suggestive of an enhanced capacity for expression of the neuroprotective and synapse-strengthening functions of this signaling system. However, the overall lack of significant stress or interaction effects implies that the observed differences in hippocampal *Bdnf* and *Trkb* mRNA expression are not likely to provide the key neural substrate(s) directly mediating differential stress response habituation in exercised and sedentary rats.

### Conclusions

This study provided a starting point for the elucidation of neurochemical mechanisms potentially underlying exercise-induced facilitation of glucocorticoid and cardiovascular response habituation to repeated audiogenic stress exposures that were recently reported (Sasse et al., 2008; Nyhuis et al., 2010; Masini et al., 2011). The primary conclusion that can be drawn from these results is that the brains of sedentary and physically active animals respond in multiple different ways to acute and repeated stress exposures. It is likely that some of the central changes reported here mediate various exercise-induced physiological adaptations (i.e., metabolic regulation). Additional direct intervention studies will be required to determine whether or how these observed brain modifications contribute to the enhanced rate of stress response habituation observed in exercised animals. Because it is the situations involving prolonged or repeated stress that are most strongly associated with illness, the possibility that interventions as cost-effective as regular exercise could enhance the rate of adaptation of potentially deleterious physiological responses to such situations strongly indicates the need for further investigation.

## Author contributions

Sarah K. Sasse performed the animal experiment with significant technical contributions from Tara J. Nyhuis, Cher V. Masini, Heidi E. W. Day and Serge Campeau. All *in situ* hybridization histochemistry was conducted by Sarah K. Sasse and Tara J. Nyhuis. Data analysis was performed by Sarah K. Sasse and interpreted by Sarah K. Sasse and Serge Campeau. Sarah K. Sasse and Serge Campeau wrote the manuscript.

### Conflict of interest statement

The authors declare that the research was conducted in the absence of any commercial or financial relationships that could be construed as a potential conflict of interest.
